# Light pollution affects space use and interaction of two small mammal species irrespective of personality

**DOI:** 10.1186/s12898-019-0241-0

**Published:** 2019-06-18

**Authors:** Julia Hoffmann, Annika Schirmer, Jana Anja Eccard

**Affiliations:** Animal Ecology, University of Potsdam, Maulbeerallee 1, 14469 Potsdam, Germany

**Keywords:** Nighttime illumination, Rodents, Outdoor enclosure, Animal personality, Interspecific interactions, HIREC

## Abstract

**Background:**

Artificial light at night (ALAN) is one form of human-induced rapid environmental changes (HIREC) and is strongly interfering with natural dark–light cycles. Some personality types within a species might be better suited to cope with environmental change and therefore might be selected upon under ongoing urbanization.

**Results:**

We used LED street lamps in a large outdoor enclosure to experimentally investigate the effects of ALAN on activity patterns, movement and interaction of individuals of two species, the bank vole (*Myodes glareolus*) and the striped field mouse (*Apodemus agrarius*). We analyzed effects combined with individual boldness score. Both species reduced their activity budget during daylight hours. While under natural light conditions home ranges were larger during daylight than during nighttime, this difference vanished under ALAN. Conspecifics showed reduced home range overlap, proximity and activity synchrony when subjected to nighttime illumination. Changes in movement patterns in reaction to ALAN were not associated with differences in boldness score of individuals.

**Conclusions:**

Our results suggest that light pollution can lead to changes in movement patterns and individual interactions in small mammals. This could lead to fitness consequences on the population level.

**Electronic supplementary material:**

The online version of this article (10.1186/s12898-019-0241-0) contains supplementary material, which is available to authorized users.

## Background

In recent decades wildlife has had to cope with several different forms of human-induced rapid environmental change (HIREC), an evolutionary novel situation with more rapid change rates than experienced in the evolutionary past [1]. The animals’ response to HIREC can be divided into an initial plastic response, a learning phase to better cope with HIREC and an evolutionary response over many generations. HIREC includes habitat loss and fragmentation, the spread of exotic species, harvesting by humans, climate change and pollutants [2]. It therefore poses a great challenge for a vast majority of species and it is important to understand which characteristics of a species makes it better suited to cope with these new environmental conditions than others.

Some individuals within a species could be better suited to respond and cope with HIREC by having a certain animal personality type [3]. Thereby, if some individuals in a species have an animal personality type that enables them to cope with environmental change while the majority of individuals are unable to do so, it could lead to selection processes within the species [4]. Especially bold, aggressive and exploratory individuals are assumed to have a higher tolerance towards anthropogenic changes and thus are able to readily use human-modified landscapes in comparison to shy, less aggressive and less exploratory individuals [5, 6]. Thus, HIREC can potentially act as a strong bottleneck for certain behavioral types in a species and thereby reduce between-individual behavioral variation, which could have severe consequences for population dynamics and ecological interactions.

One HIREC that organisms face increasingly within the last decades is artificial light at night (ALAN; [7]). For millions of years organisms evolved under natural light rhythms characterized by the sun, moon and stars. Especially the change between day and night and the change in daylength throughout the seasons were a reliable predictor for seasonal changes and were used by animals and plants to synchronize to and anticipate environmental changes throughout the year [8, 9]. Animals use natural light cues to synchronize their circadian rhythm and to time important life history events such as growth, reproduction and migration [10]. However, these natural light cues are increasingly masked by artificial light at night which has the potential to disrupt a vast range of rhythms and processes in the environment [11].

Multiple studies have confirmed that light pollution, i.e. the spread of ALAN, has negative effects on a wide range of taxa including plants [12], insects [13], amphibians [14], birds [15] and mammals [16, 17], often affecting the appropriate timing of events. Some tree species react to increased ALAN by accelerating the time of bud burst [18]. Birds start singing earlier in the morning when living under light pollution [19] and small mammals change their activity pattern as an antipredatory response when subjected to artificial light as dim as moonlight [20, 21]. Several experimental and correlative studies indicate that there is a link between light pollution and the risk of tumor growth and cancer risk in humans and other animals [22].

ALAN can also change species interactions. For example, Underwood et al. could show that dogwhelks living under light pollution were less likely to seek refuge irrespective of whether a predator cue was presented or not [23]. Additionally, certain bat species can exploit the increased insect availability around street lamps while others are deterred by artificial light [24]. Meanwhile, studies on effects of ALAN on species interactions, other than predator–prey dynamics appear to be limited.

Here we study how ALAN might change interaction, i.e. coexistence or competition between individuals and between species that are belonging to the same trophic level. Further, we investigated whether responses to ALAN differ among animals with different personality types. We used two naturally co-occurring small mammal species, the bank vole (*Myodes glareolus*) and striped field mouse (*Apodemus agrarius*; [25, 26]) and studied them in a large outdoor enclosure. The two species have similar ecological requirements in habitat and diet as they inhabit fallow land, hedges and forests and are largely omnivorous [25, 26], but seem to differ in their daily activity patterns. While bank voles show a polyphasic activity pattern with prominent activity peaks during twilight [27], the few existing studies on striped field mice suggest they increase activity during the night [28]. However they have been captured during the daytime (personal observation), which suggests they may also be day active. Voles synchronize their activity phases by the use of natural light cues, especially the rising and setting of the sun [29]. Both species show consistent inter-individual differences in behavior, i.e. have a measurable animal personality [30]. Susceptibility to predation by avian and ground predators is generally high for small rodents [31] which suggests high levels of interspecific competition not only for food and space but also predator free area.

We hypothesize that both species will be affected by light pollution in regards to activity patterns, but in different ways. Bank voles may increase their activity levels during illuminated nights as they could mistake the artificial light cues for favorable twilight conditions. For striped field mice on the other hand their preference for darkness may interrupt activity under ALAN, but as the night under ALAN is still darker than daylight, their distribution of activity phases amongst day and night should remain similar as without ALAN.

Given activity and movement are highly correlated, the home ranges of both species should change accordingly. In bank voles we expect home ranges during night to increase under ALAN, since voles extend their twilight activity phases into the night [21]. For nocturnal mice we assume that under natural conditions home ranges are smaller during day than during night. Under ALAN we expect the nighttime home ranges of mice to somewhat decrease as animals might be restricted in their movement, but to still be bigger than the home ranges during the day. The general pattern of day and night home range sizes is therefore expected to be maintained in this species, but with a smaller ratio between day and night home ranges.

Individuals belonging to the same species may lose the natural light cues to synchronize their activity and should thus show reduced interaction resulting in reduced home range overlaps and reduced spatial proximity as well as reduced synchrony of activity among conspecifics. On the other hand, this masking of natural light by ALAN should lead to an increased interaction between heterospecific individuals as they are not able to use light cues appropriately to avoid competitors in time.

Small mammals typically use light as an indirect cue for predation risk [32], and bold animals may take higher risks [33], as boldness is measured by risk-taking behavior. In consequence, we expect bold individuals to have larger ranges during illuminated nights than shy individuals, or bold individuals to not decrease their home ranges due to nighttime illumination as they experience a lower perceived predation risk than shy individuals.

## Results

### Diurnality

**Fig. 1:**
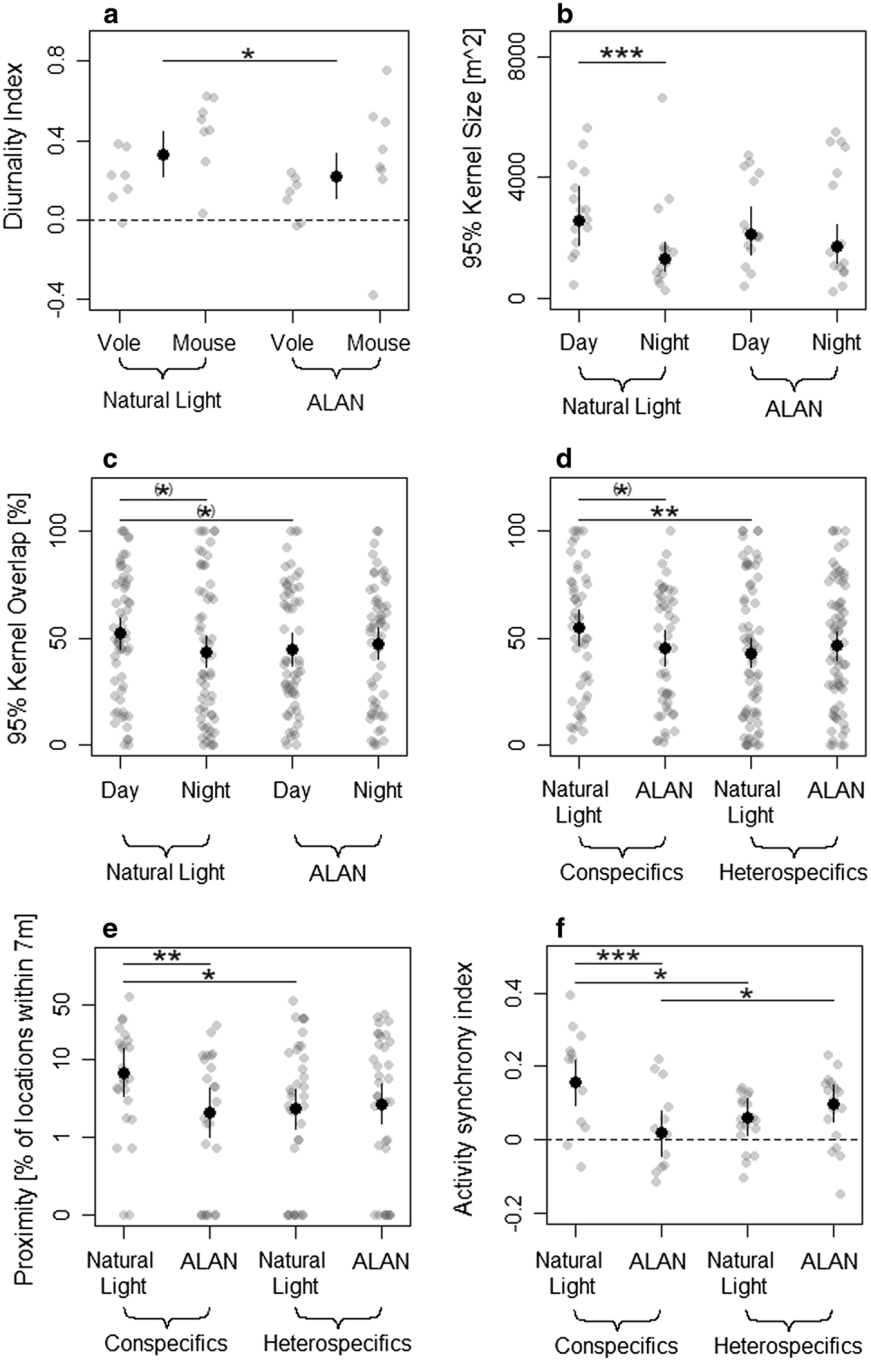
Influence of artificial light at night (ALAN) on populations consisting of two small mammal species [bank vole (*Myodes glareolus*) and striped field mouse (*Apodemus agrarius*)] in an outdoor enclosure. Grey dots show the underlying empirical data, black dots show the predicted means of the linear mixed models. Solid lines represent confidence intervals. **a** Diurnality index depending on light treatment and species. Dashed line marks the area where the diurnality index is zero and animals therefore neither prefer nor avoid daylight hours. Predicted means and confidence intervals for the main effect of light treatment are shown. The main effect of species was significant (Wald test: χ^2^ = 4.57, P = 0.032) while an interaction of both variables was not. **b** Home range size depending on the interaction of light treatment and daytime. **c** Home range overlap depending on the interaction of light treatment and daytime and **d** on the interaction of light treatment and species composition (dyads consisted of two con- or heterospecifics). **e** Proximity and **f** activity synchrony of individuals depending on light treatment and species composition. (*)—P < 0.1, *—P < 0.05, **—P < 0.01, ***—P < 0.001

On average animals of both species had positive diurnality indices of 0.28 ± 0.24 (N = 30), i.e. they preferred daylight hours over nighttime which translates into 64 ± 14% of activity being shown during daylight hours and 36 ± 14% during nighttime, respectively. The index differed between species and within animals before and after ALAN was switched on, while individual boldness score had no influence (Table 1): all animals were more active at daylight during the control period with natural light conditions (diurnality index: empirical mean 0.33 ± 0.21, N = 15) than during the time when nights were artificially illuminated (diurnality index: 0.22 ± 0.26, N = 15, Fig. 1a). Overall, striped field mice had a higher diurnality index (0.38 ± 0.27, N = 16) than bank voles (0.16 ± 0.13, N = 14), while individual boldness score had no influence on activity patterns.

**Table 1:** Overview of minimal linear mixed effects models

Dependent variable	Transformation	N	Marginal R^2^	Conditional R^2^	Fixed factor	χ^2^	P	Estimate	CI [2.5%, 97.5%]
Diurnality		30	0.244	0.709	Light	5.06	*0.025*	0.1120	[0.0113, 0.2128]
					Species	4.57	*0.032*	0.2270	[0.0284, 0.4256]
					Boldness	0.32	0.572	0.0335	[− 0.0773, 0.1443]
Home range (95% kernel)	Log	60	0.273	0.739	Light	0.13	0.721	0.1937	[− 0.1156, 0.5030]
					Daytime	15.52	*< 0.001*	− 0.2115	[− 0.5207, 0.0978]
					Species	0.29	0.592	− 0.1800	[− 0.8076, 0.4477]
					Boldness	5.13	*0.024*	− 0.4235	[− 0.7737, − 0.0734]
					Light * daytime	4.29	*0.038*	− 0.4682	[− 0.9056, − 0.0308]
Home range overlap		240	0.083	0.537	Light	0.38	0.537	0.0210	[− 0.0677, 0.1096]
					Species combination	3.02	0.083	− 0.0112	[− 0.0938, 0.0697]
					Daytime	1.06	0.303	0.0268	[− 0.0499, 0.1036]
					Boldness1	2.94	0.087	0.0604	[− 0.0215, 0.1451]
					Boldness2	0.03	0.860	− 0.0205	[− 0.1256, 0.0862]
					Light * species comb.	5.17	*0.023*	0.1297	[0.0189, 0.2404]
					Light * daytime	3.95	*0.047*	− 0.1111	[− 0.2197, − 0.0026]
					Boldness1 * boldness2	4.53	*0.033*	0.0452	[0.0042, 0.0869]
Proximity (7 m)	Log	120	0.156	0.571	Light	2.32	0.128	− 0.1548	[− 0.7809, 0.4713]
					Species combination	1.11	0.292	− 0.2608	[− 1.1874, 0.6127]
					Daytime	0.08	0.780	− 0.0700	[− 0.5550, 0.4150]
					Boldness1	0.03	0.877	− 0.1689	[− 0.7521, 0.6094]
					Boldness2	3.23	0.072	0.6026	[− 0.1571, 1.4959]
					Light * species comb.	6.88	*0.009*	1.3411	[0.3512, 2.3310]
					Boldness1 * boldness2	4.86	*0.027*	0.4396	[0.0625, 0.8297]
Activity synchrony		60	0.140	0.612	Light	2.61	0.106	− 0.0378	[− 0.0877, 0.0122]
					Species combination	0.06	0.804	− 0.0800	[− 0.1484, − 0.0112]
					Light * species comb.	18.61	*< 0.001*	0.1745	[0.0956, 0.2534]

Experimental populations of bank voles and striped field mice living under natural night conditions and under artificial light at night (ALAN) afterwards. Explained deviance of fixed factors (marginal R^2^), explained deviance of fixed factors and random effects (conditional R^2^) and results of Wald χ^2^ tests for the variables of the minimal linear mixed models are shown. Estimates and 95% confidence intervals (CI) are presented. The fixed factor light indicates the effect of a change of natural light conditions to ALAN, species the effect of bank voles compared to striped field mice, daytime the effects of daylight and nighttime, boldness the effect of the boldness score of the animals (boldness1 and boldness2 specify the boldness score of the two animals in a dyad), species combination the effect of dyads were animals are conspecifics compared to those were animals are heterospecifics. LMMs for diurnality and home range included the animal ID nested in the experimental population as a random effect. LMMs for home range overlap and proximity contained the animal ID of the focal animal and its respective opponent as well as the experimental population. Significant P values are displayed in italic

### Home range

Established home ranges (95% kernels) of animals were larger during daylight (1916 ± 1327 m^2^, N = 30) than during the night (1549 ± 1581 m^2^, N = 30). Home range size was not influenced by species but bolder animals had smaller home ranges than shyer individuals (Table 1, see Additional file 1). Additionally, we found a significant interaction between the light treatment and daytime (Table 1). The post hoc analysis revealed that under natural light conditions ranges were smaller at night (1004 ± 794 m^2^, N = 15, Table 2) than at daylight (1978 ± 1319 m^2^, N = 15, Fig. 1b) but did not differ between daylight and night when ALAN was switched on (daylight: 1854 ± 1379 m^2^, N = 15; night: 2095 ± 1977 m^2^, N = 15).

**Table 2:** Results of Wald χ^2^ tests

Dependent variable	Interaction	Across	Fixed	Level	χ^2^	P	Estimate
Home range	Light * daytime	Light	Daytime	Daylight	14.69	0.226	− 0.1937
				Night	29.48	0.172	0.2745
		Daytime	Light	Natural light	180.76	*< 0.001*	0.6797
				ALAN	17.50	0.186	0.2115
Kernel Overlap	Light * species combination	Light	Species combination	Conspecifics	46.25	0.063	− 0.0950
				Heterospecifics	0.92	0.337	0.0346
		Species combination	Light	Natural light	79.38	*0.010*	− 0.1185
				ALAN	0.07	0.790	0.0112
	Light * daytime	Light	Daytime	Daylight	46.14	0.063	− 0.0858
				Night	0.40	0.525	0.0253
		Daytime	Light	Natural light	45.54	0.066	0.0843
				ALAN	0.46	0.498	− 0.0268
Proximity (7 m)	Light * species combination	Light	Species combination	Conspecifics	8.97	*0.006*	− 1.1863
				Heterospecifics	0.23	0.632	0.1548
		Species combination	Light	Natural light	5.39	*0.041*	− 1.0803
				ALAN	0.31	0.575	0.2608

Post-hoc analysis for significant interactions in the minimal LMMs that include categorial variables. The fixed factor light indicates the effect of a change of natural light conditions to artificial light at night (ALAN), daytime the effects of daylight and nighttime, species composition the effect of dyads were animals are conspecifics compared to those were animals are heterospecifics. The significance level was adjusted for multiple testing according to Holm. Significant P values are displayed in italic

### Home range overlap

Averaged over all possible inter- and intraspecific dyads (N = 240), the home range (95% kernel) overlap amounted to 47 ± 30%. Interactions of light treatment and species combination within the dyad, as well as light treatment and daytime and the interaction of the boldness score of both animals involved were significantly influencing home range overlap (Table 1). During the experimental phase with natural light conditions, overlap was significantly higher in conspecific dyads (54 ± 30%, N = 48) than in heterospecific dyads (44 ± 32%, N = 72, Table 2, Fig. 1d). Home range overlaps in conspecific dyads tended to decrease when subjected to ALAN (44 ± 28%, N = 48) compared to overlaps under natural light conditions (54 ± 30%, N = 48), which was not the case in heterospecific dyads.

Irrespective of species combination, under natural light conditions home range overlaps tended to be larger during daytime (52 ± 30%, N = 60) than during nighttime (43 ± 34%, N = 60, Table 2, Fig. 1c). The daytime overlaps tended to be larger when animals experienced natural light conditions at night (52 ± 30%, N = 60) compared to ALAN (44 ± 29%, N = 60), while during night overlaps did not differ between light treatments (control: 43 ± 34%, N = 60; ALAN: 47 ± 28%, N = 60).

Additionally, the boldness score of the two animals in a dyad affected the overlap: with increasing boldness of the focal animal, the increase in home range overlap was stronger if the other individual was a bold animal in comparison to if it was a shy animal (see Additional file 2). Shy individuals had a higher overlap with other shy individuals, while bold animals overlapped more with other bold animals.

### Proximity

On average, individuals of a dyad were proximal (distance threshold = 7 m) 9 ± 12% of the simultaneous location fixes (N = 120). Proximity was not influenced by daytime but an influence of an interaction of light treatment and species combination as well as of an interaction of boldness score of both individuals could be observed (Table 1). Under control conditions proximity was higher in a conspecific dyad (empirical mean: 12 ± 15%, N = 24) than between heterospecific individuals (9 ± 14%, N = 36, Fig. 1e). This difference was not present under artificial light at night (conspecifics: 6 ± 7%, N = 24; heterospecifics: 9 ± 11%, N = 36). In a dyad consisting of heterospecifics the percentage of proximate fixes did not change between natural nighttime light conditions (9 ± 14%, N = 36) and ALAN (9 ± 11%, N = 36, Table 2). Meanwhile, it decreased in dyads composed of two conspecifics (natural night light: 12 ± 15%, N = 24; ALAN: 6 ± 7%, N = 24).

Boldness score of both individuals in a dyad affected their proximity. With increasing boldness of one individual of the dyad the percentage of proximal fixes decreased when the other individual was shy and increased when it was bold (see Additional file 3). Shy individuals share a similar proximity with individuals of differing boldness types. In bold individuals the percentage of proximal fixes with other bold individuals was high, but low with other shy individuals.

### Activity synchrony

Averaged over all dyads, the index of activity synchrony amounted to 0.08 ± 0.12 (N = 60). Synchrony was influenced by an interaction of light treatment and species combination but not by an interaction of the boldness scores of both individuals of the dyad (Table 1). Under natural light conditions synchrony in activity was higher in conspecifics (0.17 ± 0.14, N = 12, Table 2, Fig. 1f) than in heterospecifics (0.05 ± 0.07, N = 18) while under ALAN it was lower in conspecifics (0.03 ± 0.12, N = 12) than in heterospecifics (0.09 ± 0.10, N = 18). In a dyad consisting of conspecifics synchrony was higher under natural light conditions (0.17 ± 0.14, N = 12) than under ALAN (0.03 ± 0.12, N = 12). This was not the case in heterospecific dyads (natural light: 0.05 ± 0.07, N = 18, ALAN: 0.09 ± 0.10, N = 18).

## Discussion

Artificial light at night (ALAN) changed the behavior of two small mammal species in several ways. When night hours were illuminated, animals decreased their activity share during daylight. Furthermore, home ranges under ALAN conditions were not reduced at night in comparison to daylight ranges while space use clearly differed between daylight and night under natural light conditions. Home range overlap, proximity and activity synchrony of conspecifics were reduced during ALAN.

Our results suggest that small mammals immediately react to the sudden appearance of artificial nighttime illumination adjusting their activity pattern, space use, and interaction. When subjected to artificial light throughout the night animals of both species decreased daytime activity and synchrony of activity. While under natural light conditions home range sizes differed between daylight and night, this difference vanished under ALAN. These results indicate that ALAN is masking the natural daylight rhythm. Thus, individuals may not distinguish between daylight and night as strongly anymore. Although they would be predominantly diurnal under natural light conditions, they might shift some activity phases into the night under ALAN.

Inconsistent with other studies (e.g. [28]), we could not show that under natural light conditions striped field mice were mostly nocturnal. Instead, they had positive diurnality indices pointing to them being predominantly active at daylight. This is supported by our personal observations from live-trapping from spring to autumn, where we readily captured striped field mice during daylight trapping intervals. Bank voles like other polyphasic voles distributed their activity phases equally over day and night. Bank voles had lower diurnality indices than striped field mice, suggesting that the two sympatric species may avoid each other in time. This was supported by the reduced activity synchrony of heterospecifics compared to conspecifics under natural light conditions. Additionally, movement analysis showed that home range overlap was significantly smaller when two individuals were heterospecifics in comparison to conspecifics, thus, bank voles and striped field mice also appeared to avoid each other spatially.

Individual spatial interactions were influenced by ALAN as well. Animals belonging to the same species tended to have decreased home range overlap and decreased proximity when subjected to nighttime illumination. As natural light cues are probably masked by ALAN, individuals might not have been able to synchronize their activity phases anymore, resulting in less encounters with conspecifics. Asynchrony could have strong fitness consequences for small mammal species as establishing a territory and meeting mating partners might be more difficult if synchrony is reduced.

Neither home range size nor home range overlap were significantly influenced by the interaction of light treatment and boldness scores of individuals. This was contradicting to our expectations and suggests, that both personality types are influenced by ALAN in a similar manner. Possibly animals have similar perceptions of risk originating from the nightly illumination since predation risk is an immensely strong selection pressure for its correct judgement. Similarly, investigating the sleep behavior in great tits, Raap et al. found no influence of the personality trait exploration on the degree of sleep disruption under artificial light at night [34]. The change in conditions could be such a strong negative cue that potential effects of personality are overshadowed leading to an equal impairment by ALAN for all personality types. Under the prevalent conditions neither shy nor bold individuals may have an advantage over the other which could promote the maintenance of both types in the population.

Further, asynchronous activity reduces safety in numbers and at the same time may increase predation rate [35]. Nighttime predators may face increasingly active prey and might thus be able to use the increased illumination levels to their advantage in detecting prey individuals. Clarke [32] could already show that owls in a flight chamber were increasingly affective in hunting deer mice the more ALAN was present.

Generally, the response of animals to HIREC can be divided into three stages which consist of an initial plastic response, learning to better cope with HIREC and an evolutionary response over many generations [36]. The current study investigated the immediate reaction of animals towards a new environmental stressor. Long-term coping abilities might look very different, as even if animals did not respond well to light pollution in the beginning, they might be able to improve their ability to cope later on. Nevertheless, we already could show in another experiment, that very dim but long-term ALAN has the potential to cause long-term behavioral changes regarding activity and space use in rodents [21]. Future studies should investigate the evolutionary responses of animals to ALAN, as knowledge on this topic is quite scarce (but see [37]).

## Conclusion

As one type of human-induced rapid environmental change connected to urbanization light pollution is increasingly affecting animals by leading to changes in multiple aspects of their behavior. Here we show that interactions of coexisting species on the same trophic level were altered by ALAN. The species probably undergo increased competition and interference, since temporal and spatial avoidance patterns were disturbed. Similarly, animals may face increased predation risk if their conspecific activity cycles are becoming desynchronized by ALAN. Together with a loss of synchrony in mate search and territory defense, ALAN may have fitness consequences on the local population level.

## Materials and methods

### Study subjects and experimental site

The study was conducted from August to October 2017 in grassland outdoor enclosures near Potsdam, Eastern Germany. Adult bank voles and striped field mice were wild-captured in August and September 2017. Individuals were kept in standard rodent cages on a standard rodent diet until the experiment for 27 days on average. For individual identification they were equipped with a passive integrated transponder tag (PIT; trovan ID-100, 2.12 mm × 11.5 mm, 0.1 g).

The experiment was conducted in a large outdoor enclosure with a size of 0.25 ha (50 × 50 m). The enclosure was surrounded by a galvanized metal wall extending 1 m below and 0.5 m above ground. An electrical veterinary fence surrounding the experimental facility protected study animals against larger terrestrial predators while enclosures were open to avian predation. Multicapture live traps (Ugglan special No2, Grahnab, Sweden) were evenly distributed across the enclosure (N = 25, 5 × 5 trapping grid with 10 m distance between traps).

### Experimental design

Bank voles and striped field mice were transferred to the enclosure in three consecutive rounds in August, September and October 2017. Each of the three experimental small mammal populations initially consisted of four bank voles and four striped field mice with two males and two females per species. Animals lived under natural light conditions for 6 to 7 days before the ALAN treatment began. Due to predation events and partial failing of radio collars we analyzed before-after data of 15 animals (5 animals per round), seven bank voles and eight striped field mice within the three experimental populations, producing 48 intra- and 72 interspecific dyadic interactions of individuals. In the three consecutive rounds we radio tracked two, three and two bank voles and three, two and three striped field mice, respectively, with the same absolute density of individuals in each round.

For the ALAN treatment we used four LED street lamps (Schréder TECEO 1, 32 LEDs 500 mA, Optic 5103). Each lamp head was mounted on a rack (height 4 m) and tilted upwards by 10°. The street lamps generated a “warm white” light through 32 diodes (color temperature = 3000 K, for spectral properties see Additional file 4). Lamps were programmed to switch on at sunset and off at sunrise. Illuminance was measured at ground level at all 25 trapping positions and ranged from > 0.1 lx to 38.6 lx (Extech HD450, measuring range 0.1–400,000 lx). Mean illuminance was 5.8 lx. The study animals were subjected to ALAN for four to five nights. Then, they were captured from the enclosure and were returned to the laboratory.

### Test for individual differences

Animals were tested for consistent inter-individual differences prior to the experiment using a standardized behavioral test. Animals entered the test twice with 1 week between the test rounds. The setup combines the dark–light-test and the open-field-test, which both are established tests for measuring behavioral differences in rodents [38, 39]. In short, animals were first observed when leaving a pipe attached to an arena. Once the individual entered the arena, the latency to enter the middle area, the number of crossings of the middle area and activity when exploring the open field were quantified. Boldness was expressed as a combined score of the latency to stick the head out of the pipe and the latency to leave the pipe with the whole body. The test procedure is described in detail in [40], measuring variables to categorize individuals concerning for boldness and exploration. We concentrated on boldness as the personality trait that is likely of importance in relation to ALAN, as light is typically seen as an indirect cue of predation risk in small mammals [32, 41, 42] and boldness is connected to mortality risk and survival in many species [33].

### Activity and space use

We conducted VHF radio telemetry using an automated radio telemetry system, consisting of four omnidirectional antennae (GP 150 Winkler-Spezialantennen, Annaberg, Germany). Antennas were connected to an automatic receiving unit (ARU; Sparrow System, USA) which logged signal strength per frequency and antenna. Each antenna was mounted on a rack in a corner of the enclosure (height: 1.5 m) and signals were transmitted to the ARU by underground cables. Study animals were equipped with radio telemetry transmitters (BD-2C, Holohil Systems Ltd., 1.1 g) prior to the experiment. Ratio of transmitter to study animal body weight did not exceed 0.05. The ARU scanned for each radio frequency every 5 min for seven times per antenna within a period of 24 s before switching to the next radio frequency.

Activity patterns were analyzed by using signal strength variation between subsequent logged signals. A change in position or posture of the animal is thereby indicated through a large absolute difference in signal strength. We used a transmitter-specific absolute threshold of changes in signal strength to define animals as active. The threshold method robustly reveals the same individual activity pattern across a range of transmitter-specific thresholds. We defined a transmitter specific threshold where 25% of the highest differences in signal strength were defined as active. This value is in accordance with reports on bank vole activity (< 20% [43]; > 25% [44]). However, with this method we can not analyze differences in the total amount of activity between animals, but the distribution of activity over the day.

A diurnality index proposed by Halle [45, cf. 46] was calculated for a 24 h-period before and after nighttime illumination was switched on. The index I_D_ is calculated as follows:

$$I_{D} = \left( {\frac{{\frac{\varSigma cD}{hD}}}{{\frac{\varSigma cD}{hD} + \frac{\varSigma cN}{hN}}}} \right)*2 - 1$$

where ∑cD and ∑cN are the activity counts during day (from sunrise to sunset) and night (from sunset to sunrise), respectively. The terms hD and hN describe day length and night length. The index ranges from − 1 to + 1 and is positive when an animal is predominantly active during the day.

We analyzed the synchrony in activity of individuals within each dyad before and after nighttime illumination. We used an index proposed by Michelena et al. [47] which is an adaptation of the coefficient of association r_ϕ_ [48]. The index r_ϕ_ was computed as follows:

$$r_{\phi } = \frac{A*D - B*C}{{\sqrt {\left( {A + B} \right)*\left( {C + D} \right)*\left( {A + C} \right)*\left( {B + D} \right)} }}$$

where A and D are the times during which both individuals of a dyad where classified as active or inactive, respectively. The terms B and C describe the time during which individual 1 is active while individual 2 is inactive and vice versa. Similar to the diurnality index, the synchrony index can range from − 1 to + 1. Positive values indicate a synchronization in activity, while negative values indicate that individuals of a dyad are not simultaneously active or inactive.

The locations were calculated using the median signal strengths of seven scans per antenna, resulting in 288 locations per animal in 24 h. We used the relative spatial distribution of logged signal strengths to obtain cartesian coordinates through isolines in x and y direction, respectively. Equations for isolines were calibrated using signal strengths for 22 known positions of calibration transmitters in the enclosure. The obtained locations had some deviation from the true location of an animal (deviation D_X_ = 7.8 ± 7.1 m, D_Y_ = 7.1 ± 6.1 m). Potential grid distortions are taken care of by using a repeated measures study design with comparisons within each telemetry grid.

With the obtained coordinates we estimated for each individual the day and night ranges using fixed kernels containing 95% of all positions. We compared a 24 h time window before and after ALAN was switched on. For the analysis of spatial interaction of individuals, we investigated a static component (home range overlap) and a dynamic component (proximity) for each dyad. We determined the home range overlap of the estimated 95% kernels. Furthermore, we analyzed the proximity of individuals, i.e. the percentage of time the individuals within each possible dyad were close to one another. We defined two animals as proximal if they were less than seven meters apart within the same tracking interval, based on potential deviations within the tracking system. Preliminary analysis of other thresholds to define proximity yielded very similar results.

### Statistical analyses

All statistical analyses were done with R 3.5.0 [49] and for each analyzed variable we present the mean together with the standard deviation.

We built linear mixed models (LMMs) using the R package lme4 [50] to analyze the effects of the light treatment on the variables diurnality, home range size (kernels), home range overlap and proximity. As sample sizes of all analyzed dependent variables differ from each other, a model of different complexity, i.e. containing a different set of additional fixed factors, was built for each of the variables (see Additional file 5). The full model to analyze the diurnality index (N = 30 animal days) contained an interaction of light treatment and species as well as the variable boldness score as fixed factors. To analyze home range size (N = 60 daylight and night ranges), a model consisting of an interaction of light treatment and daytime (daylight and night), an interaction of light treatment and boldness score and the variable species. The model to analyze home range overlap (N = 240 overlaps of daylight and night ranges) contained an interaction of light treatment and species combination (individuals of a dyad can either be con- or heterospecific), an interaction of light treatment and daytime, and an interaction of light treatment and the boldness scores of both individuals that constitute the dyad. Proximity (N = 120 dyads during daylight and night) was analyzed using a full model with the interactions light treatment and species combination, light treatment and daytime, and boldness score of the first and second individual of the dyad. The full model to analyze activity synchrony (N = 60) consisted of an interaction of light treatment and species combination and an interaction of the boldness scores of both individuals of the dyad. The variables home range size and proximity were transformed logarithmically to fit a normal distribution. We tried to incorporate an interaction of boldness differences within a dyad of animals and light treatment into the models regarding home range overlap and proximity but this reduced the explanatory power of the models drastically. Therefore, we rather included an interaction of each of the boldness scores of the two animals of a dyad. As our sample size was small, we could not include sex as a fixed factor in the models. Based on our earlier results on free-ranging animals of the same species in mixed communities [40, 51] we do not expect differences in movement and space use between females and males.

As the experiment includes repeated measurements of the same individual, a random term for the individual was included into each model. Additionally, we included the experimental population as a random term to account for differences in species distribution and changes in weather conditions. LMMs for diurnality and home range included the individual nested in the experimental population while LMMs for home range overlap and proximity contained the both individuals of the dyad and the experimental population (see Additional file 5). To confirm a regular error distribution, we plotted residuals versus fitted values and Q–Q-plots. Full models were then reduced via stepwise backwards selection and by comparing the Akaike Information Criterion (AIC). Dependent variables that were part of the experimental setup or hypotheses such as light treatment, species and daytime were never excluded from the models (see Additional file 5 for full and reduced model per variable). We assessed the explained deviance of the most parsimonious model for the fixed effects alone (marginal R^2^) and fixed effects and random effects together (conditional R^2^) according to Nakagawa and Schielzeth [52].

Significance of fixed factors in the minimal model was assessed by a Wald test (χ^2^). Due to the small sample size in our study we refrained from conducting statistical correction for multiple testing since it would increase the probability of type-II errors to high levels [53]. Further analyses of significant interactions were conducted using the R package phia [54]. Simple main effects for interactions were analyzed by evaluating the contrasts across the levels of one interaction factor while the values of the other factor were fixed. The significance level was adjusted for multiple testing according to Holm.

## Additional files

**Additional file 1.** Influence of boldness score on home range size (95 % kernel). Grey dots show the underlying data. The solid line represents the prediction line from the linear mixed effects model, dashed lines represent confidence intervals. The higher the boldness score, the bolder is the animal.

**Additional file 2.** Home range overlap depending on boldness scores of both individuals of a dyad. Prediction lines from linear mixed models are shown.

**Additional file 3.** Proximity depending on boldness scores of both individuals of a dyad. Prediction lines from linear mixed models are shown.

**Additional file 4.** Spectral properties of LED street lights used in the experiment. Spectral properties were measured by the Ferdinand-Braun-Institut, Leibniz-Institut fuer Hoechstfrequenztechnik (FBH).

**Additional file 5.** Linear mixed models (LMMs) before (full) and after (minimal) model simplification. The fixed factor light indicates the effect of a change of natural light conditions to ALAN, species the effect of bank voles compared to striped field mice, daytime the effects of daylight and nighttime, boldness the effect of the boldness score of the animals (boldness1 and boldness2 specify the boldness score of the two animals in a dyad), species composition the effect of dyads where animals are conspecifics compared to those were animals are heterospecifics. LMMs for diurnality and home range included the animal ID nested in the experimental population as a random effect. LMMs for home range overlap, proximity and activity synchrony contained the animal ID of both individuals of the dyad (ID1 and ID2) as well as the experimental population (Population) as random effects.

## Data Availability

The datasets used and analyzed during the current study are available from the corresponding author on reasonable request.

## Abbreviations

ALAN: artificial light at night
ARU: automatic receiving unit
HIREC: human-induced rapid environmental change
LED: light-emitting diode
LMM: linear mixed model
