# Supplementary figures and images for: Light pollution affects space use and interaction of two small mammal species irrespective of personality
Source: BMC Ecol. 2019 Jun 18;19:26. doi: 10.1186/s12898-019-0241-0 (PMC6582560; doi:10.1186/s12898-019-0241-0)

**Additional file 1**

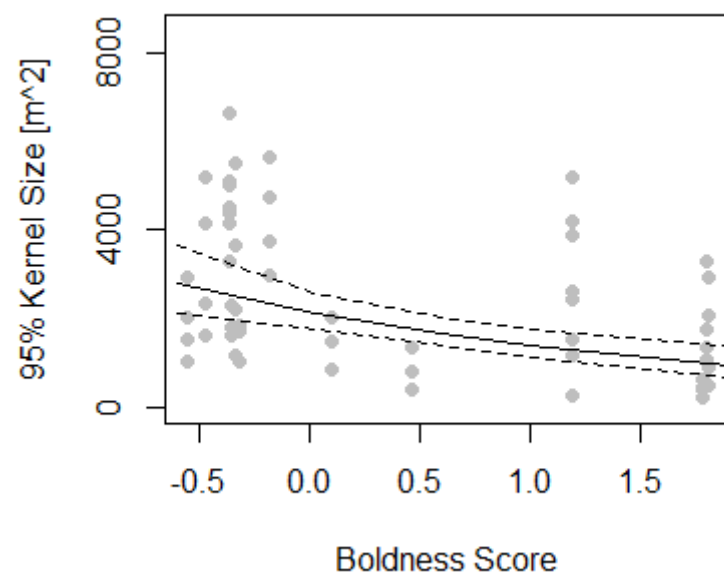

Supplement: Supplementary file 1 — Additional file 1. Influence of boldness score on home range size (95 % kernel). Grey dots show the underlying data. The solid line represents the prediction line from the linear mixed effects model, dashed lines represent confidence intervals. The higher the boldness score, the bolder is the animal. [file 12898_2019_241_MOESM1_ESM.pdf]

## Additional file 2

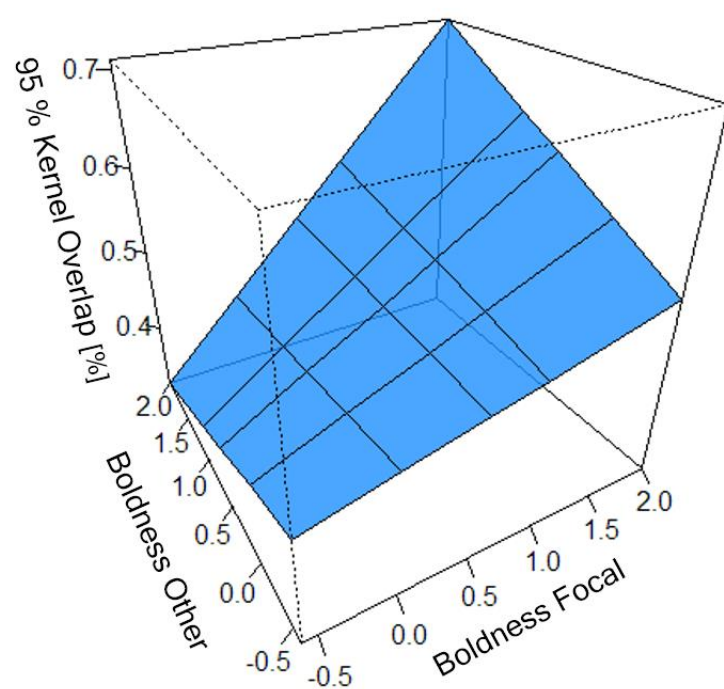

Supplement: Supplementary file 2 — Additional file 2. Home range overlap depending on boldness scores of both individuals of a dyad. Prediction lines from linear mixed models are shown. [file 12898_2019_241_MOESM2_ESM.pdf]

### Additional file 3

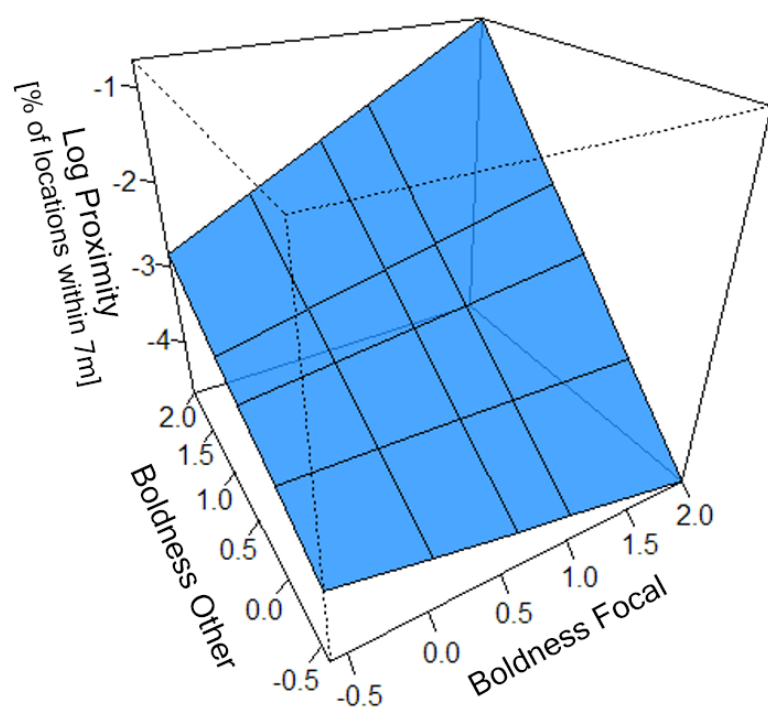

Supplement: Supplementary file 3 — Additional file 3. Proximity depending on boldness scores of both individuals of a dyad. Prediction lines from linear mixed models are shown. [file 12898_2019_241_MOESM3_ESM.pdf]

#### Additional file 4

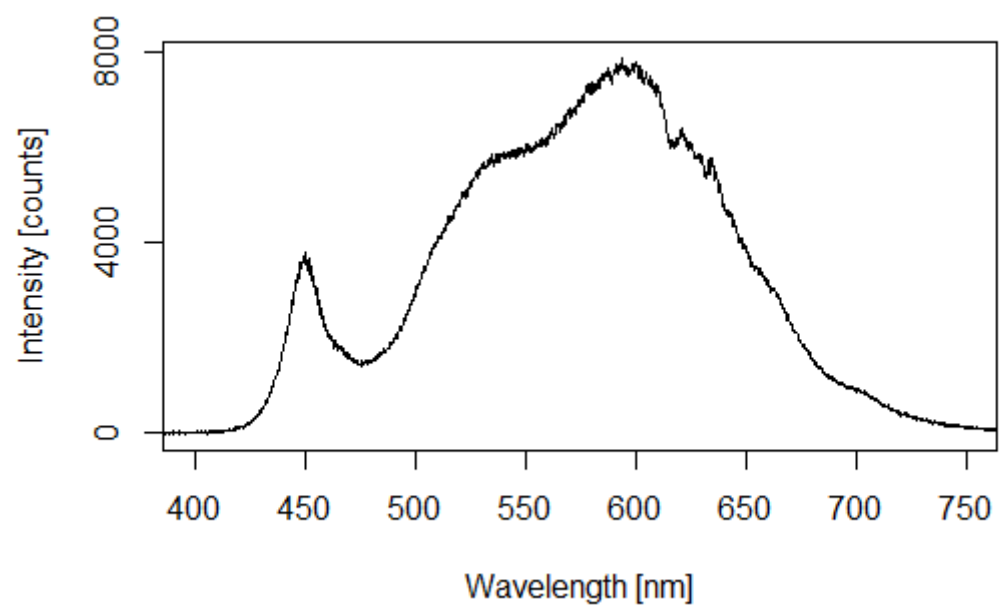

Supplement: Supplementary file 4 — Additional file 4. Spectral properties of LED street lights used in the experiment. Spectral properties were measured by the Ferdinand-Braun-Institut, Leibniz-Institut fuer Hoechstfrequenztechnik (FBH). [file 12898_2019_241_MOESM4_ESM.pdf]
