# Supplementary material for: Light pollution affects space use and interaction of two small mammal species irrespective of personality
Source: BMC Ecol. 2019 Jun 18;19:26. doi: 10.1186/s12898-019-0241-0 (PMC6582560; doi:10.1186/s12898-019-0241-0)
Supplement: Supplementary file 5 — Additional file 5. Linear mixed models (LMMs) before (full) and after (minimal) model simplification. The fixed factor light indicates the effect of a change of natural light conditions to ALAN, species the effect of bank voles compared to striped field mice, daytime the effects of daylight and nighttime, boldness the effect of the boldness score of the animals (boldness1 and boldness2 specify the boldness score of the two animals in a dyad), species composition the effect of dyads where animals are conspecifics compared to those were animals are heterospecifics. LMMs for diurnality and home range included the animal ID nested in the experimental population as a random effect. LMMs for home range overlap, proximity and activity synchrony contained the animal ID of both individuals of the dyad (ID1 and ID2) as well as the experimental population (Population) as random effects. [file 12898_2019_241_MOESM5_ESM.pdf]

Additional file 5

Dependent variable	N	Transformation	Model complexity	Fixed factors	Random effects	AIC
Diurnality	30		Full	Light * Species + Boldness	Population/ID	-1
			Minimal	Light + Species + Boldness	Population /ID	-3
Home range (95 % kernel)	60	Log	Full	Light * Daytime + Light * Boldness + Species	Population /ID	116
			Minimal	Light * Daytime + Boldness + Species	Population /ID	114
Home range overlap	240		Full	Light * Species combination + Light * Daytime + Light * Boldness1 * Boldness2	ID1 + ID2 + Population	28
			Minimal	Light * Species combination + Light * Daytime + Boldness 1 * Boldness2	ID1 + ID2 + Population	23
Proximity 5m	120	Log	Full	Light * Species combination + Light * Daytime + Boldness1 * Boldness2	ID1 + ID2 + Population	457
			Minimal	Light * Species combination + Daytime + Boldness1 * Boldness2	ID1 + ID2 + Population	455
Proximity 6m	120	Log	Full	Light * Species combination + Light * Daytime + Boldness1 * Boldness2	ID1 + ID2 + Population	463
			Minimal	Light * Species combination + Daytime + Boldness1 * Boldness2	ID1 + ID2 + Population	461
Proximity 7m	120	Log	Full	Light * Species combination + Light * Daytime + Boldness1 * Boldness2	ID1 + ID2 + Population	465
			Minimal	Light * Species combination + Daytime + Boldness1 * Boldness2	ID1 + ID2 + Population	463
Activity synchrony	60		Full	Light * Species combination + Boldness1 * Boldness 2	ID1 + ID2 + Population	-93

	Minimal	Light * Species combination	ID1 + ID2 + Population	-97
